# Weak Metal–Support Interaction over CuO/TiO_2_ Catalyst Governed Low-Temperature Toluene Oxidation

**DOI:** 10.3390/nano13121859

**Published:** 2023-06-14

**Authors:** Meilin Zou, Mingyue Wang, Jingge Wang, Danrui Zhu, Jiaying Liu, Junwei Wang, Qingchao Xiao, Jianjun Chen

**Affiliations:** 1 Faculty of Environment Science and Engineering, Kunming University of Science and Technology, Kunming 650500, China; zoumeilin@stu.kust.edu.cn (M.Z.); wangmingyue@stu.kust.edu.cn (M.W.); wangjingge@stu.kust.edu.cn (J.W.); zhudanrui@stu.kust.edu.cn (D.Z.); liujiaying@stu.kust.edu.cn (J.L.); wangjunwei@stu.kust.edu.cn (J.W.); 2 Kunming Youdu Environmental Monitoring Co., Ltd., Kunming 650100, China; lock_sleeping@163.com

**Keywords:** weak metal–support interaction, CuO/TiO_2_, toluene catalytic oxidation

## Abstract

Regulating the metal–support interaction is essential for obtaining highly efficient catalysts for the catalytic oxidation of volatile organic compounds (VOCs). In this work, CuO-TiO_2_(coll) and CuO/TiO_2_(imp) with different metal–support interactions were prepared via colloidal and impregnation methods, respectively. The results demonstrated that CuO/TiO_2_(imp) has higher low-temperature catalytic activity, with a 50% removal of toluene at 170 °C compared to CuO-TiO_2_(coll). Additionally, the normalized reaction rate (6.4 × 10^−6^ mol·g^−1^·s^−1^) at 160 °C over CuO/TiO_2_(imp) was almost four-fold higher than that over CuO-TiO_2_(coll) (1.5 × 10^−6^ mol·g^−1^·s^−1^), and the apparent activation energy value (27.9 ± 2.9 kJ·mol^−1^) was lower. Systematic structure and surface analysis results disclosed that abundant Cu^2+^ active species and numerous small CuO particles were presented over CuO/TiO_2_(imp). Owing to the weak interaction of CuO and TiO_2_ in this optimized catalyst, the concentration of reducible oxygen species associated with the superior redox property could be enhanced, thus significantly contributing to its low-temperature catalytic activity for toluene oxidation. This work is helpful in exploring the influence of metal–support interaction on the catalytic oxidation of VOCs and developing low-temperature catalysts for VOCs catalytic oxidation.

## 1. Introduction

Volatile organic compounds (VOCs) have various kinds and come from a wide range of sources, mainly including but not limited to chemical industries, transportation, household products and office supplies [1]. As an important precursor to the formation of PM_2.5_ and O_3_ [2,3,4], VOCs are prone to photochemical reactions that produce photochemical smog, which is currently the focus of air pollution control. At present, the purification methods for volatile organic compounds include the catalytic combustion, absorption and adsorption, biological and photocatalysis methods [5]. Among these methods, catalytic combustion has the advantages of high removal efficiency, less secondary pollution and low energy consumption, making it one of the most effective methods for removing VOCs [1,6,7] that is widely used. However, the exhaust gas temperature is low in practical applications; thus, further research is needed on the low-temperature catalytic oxidation of VOCs. The core of catalytic combustion method is catalysts, mainly including two types of catalysts: supported noble metal catalysts and transition metal oxides catalysts [8]. The key challenge in the development and progress of catalytic combustion methods is how to design and prepare catalysts with excellent catalytic activity, stability and universality. 

Precious metal-based catalysts usually have the advantages of high activity and good stability, typically Pd, Pt, Rh etc. [9,10,11,12]. However, the scarcity and high price heavily limit the widespread use of noble metal catalysts. In recent years, transition metal-based catalysts became a research focus in this field due to low energy consumption, abundant resources, being much cheaper than precious metals [13] and the fact that some metal oxide catalysts show excellent catalytic activity and stability. The valence state, specific surface area [14] and porous structure of the metal will affect the catalytic performance of the catalyst. In addition, the influence of support on transition metal oxides was often studied [15], and composite transition metal catalysts became a research hotspot due to synergistic effects [16]. Numerous transition metal oxides are used in catalytic combustion of VOCs, mainly including cobalt (Co), iron (Fe), copper (Cu), cerium (Ce) and manganese (Mn) [17]. Among them, copper and its oxides are widely studied, mainly because of the high redox potential, environment friendly nature, the existence of earth-abundant reserves and the low cost of use [18]. M. Konsolakis et al. [15] explored the effects of a series of Rare Earth Oxide(s) (REO) supports (CeO_2_, Gd_2_O_3_, La_2_O_3_ and Sm_2_O_3_) from Cu-based catalysts on ethyl acetate (EtOAc) oxide. The results showed that the supports had significant impacts on the redox properties of Cu/REO catalysts, thereby affecting their VOC oxidation ability. Among them, Cu/CeO_2_ samples displayed the superior catalytic performance, achieving complete conversion of EtOAc at 275 °C. Additionally, CuMn bimetallic oxides were also widely used for catalytic oxidation of toluene. A series of CuMn bimetallic oxides for the toluene catalytic oxidation were synthesized by S. Xiong et al. [19], and they found that the composite of Cu and Mn significantly increases the Brunauer–Emmett–Teller (BET) specific surface area, resulting in an increase in toluene adsorption capacity, thereby improving the catalytic activity. Among the CuMn catalysts, Cu_2_Mn_1_ had the highest BET surface areas (82 m^2^·g^−1^), which promotes the most excellent oxidation activity with 90% conversion of toluene at 224 °C (T_90_ = 224 °C). In order to further study the catalytic performance of composite metal oxides, CuO-CeO_2_ catalyst is constructed. Y. Zeng et al. [20] synthesized highly active CuO-CeO_2_ (CuCe-DR) catalyst via a novel double redox (DR) method for catalytic oxidative decomposition of toluene. They found that the moderate Cu–Ce interaction can promote the electron transfer between CuO and CeO_2_, thereby improving the redox performance of CuCe-DR catalyst. 

Titanium dioxide (TiO_2_) is not only widely applied to photocatalytic technology, but is also one of the most frequently used supports for metal oxide catalysts, having the characteristics of excellent electron transfer properties, high chemical stability and an environmentally benign nature [21,22,23]. In the meantime, TiO_2_ also exhibits catalytic performance for pollutants, while composites TiO_2_ and CuO could enhance the catalytic performance [18]. Y. Fang et al. [24] constructed an extensive Cu, incorporating TiO_2_-induced nucleophilic oxygen structures in the CuTiO_x_ catalyst, and found that it showed superior catalytic activity at low temperatures in C_3_H_6_ oxidation with the 90% conversion rate at 212 °C. However, the extent of the effect of the interaction on Cu–Ti interaction on toluene oxidation remain an open question. 

In present work, two catalysts of CuO-TiO_2_(coll) and CuO/TiO_2_(imp) with different metal–support interactions were constructed. The physicochemical properties of the catalysts were systematically characterized via N_2_ adsorption–desorption analyses, X-ray diffraction (XRD), Laser Micro-Raman spectra, scanning electron microscope (SEM), transmission electron microscopy (TEM), X-ray photoelectron spectra (XPS), ultraviolet-visible spectroscopy (UV-vis) and H_2_ temperature programmed reduction (H_2_-TPR). The relationship between metal–support interaction and catalytic performance was disclosed.

## 2. Materials and Methods

### 2.1. Chemical

Cu(NO_3_)_2_·3H_2_O, titanium tetraisopropanol, 1-butanol, HNO_3_ and deionized water were used in this study. Cu(NO_3_)_2_·3H_2_O and 1-butanol were obtained from Sinopharm Chemical Reagent Co., Ltd. (Shanghai, China). Titanium tetraisopropanol (97%) and HNO_3_ were purchased from Sigma Aldrich Trading Co., Ltd. (Shanghai, China) and Chengdu Chron Chemical Co., Ltd. (Chengdu, China), respectively. All the reagents were analytically pure (AR grade) and were used in this work without further purification.

### 2.2. Catalyst Preparation

#### 2.2.1. The Synthesis of CuO-TiO_2_(coll) and TiO_2_

The CuO-TiO_2_(coll) and TiO_2_ were synthesized via a similar approach. The synthesis of CuO-TiO_2_(coll) catalyst began with adding 9.5 mL 1-butanol solution and 4.9 mL titanium tetraisopropyl alcohol to the beeper, and 0.2278 g Cu(NO_3_)_2_·3H_2_O was then added to dissolve via stirring at room temperature. Next, 2.5 mL HNO_3_ was added drop by drop under vigorous stirring, heated in a water bath, and stirred at 70 °C to the yellow–green gel. Finally, the gel was dried overnight at 80 °C and calcined at 450 °C for 4 h with a heating rate of 1 °C·min^−1^ in air.

The TiO_2_ powder was synthesized for use as a support. Firstly, 5.5 mL 1-butanol and 18 mL titanium tetraisopropyl alcohol were added into the three-neck flask, as we stirred evenly at room temperature, and 6 mL HNO_3_ was added drop by drop under intense stirring to produce white precipitate. The condensation tube was added and stirred in a 70 °C water bath for 3 h, and the mixture was then dried at 80 °C overnight and roasted at 450 °C for 4 h.

#### 2.2.2. The Synthesis of CuO/TiO_2_(imp)

Supported catalyst CuO/TiO_2_(imp) was prepared via the excessive impregnation method. Firstly, 0.1519 g Cu(NO_3_)_2_·3H_2_O was dissolved in 0.95 mL deionized water at normal temperature, and 0.95 g TiO_2_ powder synthesized before was then added to continue stirring. The stirring procedure was 1 h at normal temperature, 1 h standing, and 1 h in water baths heated at 40 °C, 50 °C and 60 °C, respectively. Finally, the obtained powder samples were dried overnight at 80 °C and roasted at 450 °C for 4 h, and CuO/TiO_2_(imp) catalyst was obtained via pressing and sifting.

### 2.3. Catalytic Activity Test

The catalytic activities were carried out in a fixed quartz tube reactor (i.d. = 6 mm), and 300 mg of samples of 40–60 mesh were filled. Before the activity test, the catalysts were pre-treated in air at 160 °C for 30 min to remove possible impurities and moisture on the surface. The feedstock gas mixture consisted of 600 ppm toluene, which was generated via flowing dry N_2_ gas through a saturator at 0.5 °C, which was diluted with 20% O_2_ and balanced using N_2_ with a total flow rate of 100 mL·min^−1^. The weight hourly space velocity (WHSV) was 20,000 mL·g^−1^·h^−1^. The toluene concentrations in the inlet gas and outlet gas were monitored using a gas chromatograph (Fuli GC9790), which was equipped with a flame ionization detector (FID); at each reaction temperature, the gas mixture was stabilized for about 20 min. Toluene removal rate was calculated using the following Equation (1):

(1) $$\mathrm{Toluene}\;\mathrm{Removal}\;(\%)=\frac{{\left[\mathrm{Toluene}\right]}_{\mathrm{in}}-{\;[\mathrm{Toluene}]}_{\mathrm{out}}}{{\left[\mathrm{Toluene}\right]}_{\mathrm{out}}}\;\times \;100\%$$

where [Toluene]_in_ represents the concentration of toluene in the inlet gas and the [Toluene]_out_ represents the concentration of toluene in the outlet gas from the reactor. T_50_ and T_90_ correspond to the reaction temperature when the toluene removal rate is 90% and 50%, respectively.

The reaction rate r was defined using the Equation (2):

(2) $$r\;\left(\mathrm{mol}\cdot g^{-1}\cdot s^{-1}\right)=\frac{X_{\mathrm{Touluene}}\cdot V_{\mathrm{Toluene}}}{m_{\mathrm{cat}}\cdot V_{m}\cdot 3600}$$

where X_Toluene_ is the inversion rate of toluene, V_Toluene_ is the inlet volumetric flow of Toluene (L·h^−1^), m_cat_ is the mass of catalysts (m_cat_ = 0.3 g) and V_m_ = 22.4 L·mol^−1^.

### 2.4. Kinetic Analysis

The kinetic study was conducted under the condition of 75,000 mL·g^−1^·h^−1^ and 80 mg samples. The inlet gaseous concentration of toluene was set at 600 ppm in order to keep the toluene removal rate below 15%, where the effect of heat and mass transfer was negligible [25]. The activation energy (E_a_) of toluene oxidation was calculated according to the Arrhenius equation (Equation (3)) as follows:

(3) $$\mathrm{lnr}=-\frac{E_{a}}{\mathrm{RT}}+\mathrm{lnA}$$

where r is the reaction rate ($\mathrm{mol}\cdot g^{-1}\cdot s^{-1}$), R is molar gas constant (8.314 J·mol^−1^·K^−1^), T (K) represents the reaction temperature and A is the pre-exponential factor (s^−1^).

### 2.5. Catalyst Characterization

N_2_ adsorption–desorption experiments were implemented on TriStarII3020 equipment produced by an American company. Before testing, 150 mg powder samples were weighed and pre-treated for 4 h at 300 °C under vacuum environment. Next, nitrogen adsorption and desorption were performed at −196 °C. The specific surface area of the samples was calculated using the Brunauer–Emmett–Teller (BET) formula, and the average pore size distribution was calculated via the Barrett–Joyner–Halenda model. 

Powder X-ray diffraction (XRD) patterns were performed on a Shimadzu X-ray diffractometer (XRD-6100) with Cu Kα radiation (λ = 0.15406 nm). The data of 2θ range from 5° to 85° were collected with scanning velocity of 8°/min. 

Raman spectroscopy was performed using a Renishaw Via Reflex 2000 microscopic Raman spectrometer (50×) and Leica microscopy system. The reduced samples were activated using a 532 nm solid-state laser, and the silicon wafer peak at 520.5 cm^−1^ was used to calibrate the Raman spectra before the experiment. 

The scanning electron microscope (SEM) images of the samples were obtained using the ZEISS Sigma 300 scanning electron microscope. Moreover, the scanning test was carried out using smartedx type Energy-Dispersive X-ray Spectroscopy (EDX). Transmission electron microscopy (TEM) was carried out on a Tecnai G2 F30 S-Twin device. The samples were pre-treated in ethanol solution via ultrasonically suspension, and suspension droplets were cast onto carbon-coated nickle grids and tested after ethanol volatilization. Gatan Digital Micrograph software was employed to analyze the crystal structure, particle size and morphology of the catalysts. The corresponding grain sizes of samples were estimated using Nano Measurer software. 

X-ray photoelectron spectroscopy (XPS) was conducted on Thermo Fisher Scientific ESCALAB 250Xi equipment with Al Kα (hv = 1486.6 eV), and the vacuum degree of the analysis room was 8 × 10^−10^ Pa. The binding energy of the samples was corrected using the C 1s peak of carbon at 284.8 eV. XPS quantitative analysis commonly uses the element sensitivity factor method, and the instrument used in this test corresponded to element sensitivity factors of 1 (C), 26.513 (Cu), 6.471 (Ti) and 2.881 (O), respectively. The Thermo Avantage software was used to correct the XPS data and fit the peaks, and the atomic ratio of the surface elements was then calculated according to the peak area. 

Ultraviolet-visible spectroscopy (UV-vis) was obtained using a UV-3600 Spectrophotometer (Shimadzu) in the wavelength range of 200–800 nm, which was equipped with a diffuse reflectance accessory.

The instrument used for H_2_-temperature-programmed reduction (H_2_-TPR) was a TP-5080-α automatic multi-purpose adsorption instrument. Before the test, 100 mg (20–40 mesh) samples were activated at 400 °C in N_2_ atmosphere for 30 min to remove the adsorbed water and impurities from the surface, cooled to room temperature, switched to H_2_/Ar mixture (30 mL·min^−1^), and heated to 800 °C at 8 °C/min. We checked the H_2_-TPR signal with a thermal conductivity detector, before returning to room temperature. The PeakFit software was used for peak fitting. H_2_ consumption corresponding to each peak was calculated quantitatively using standard CuO as calibration.

## 3. Result and Discussion

### 3.1. Catalytic Performance

**Figure 1:**
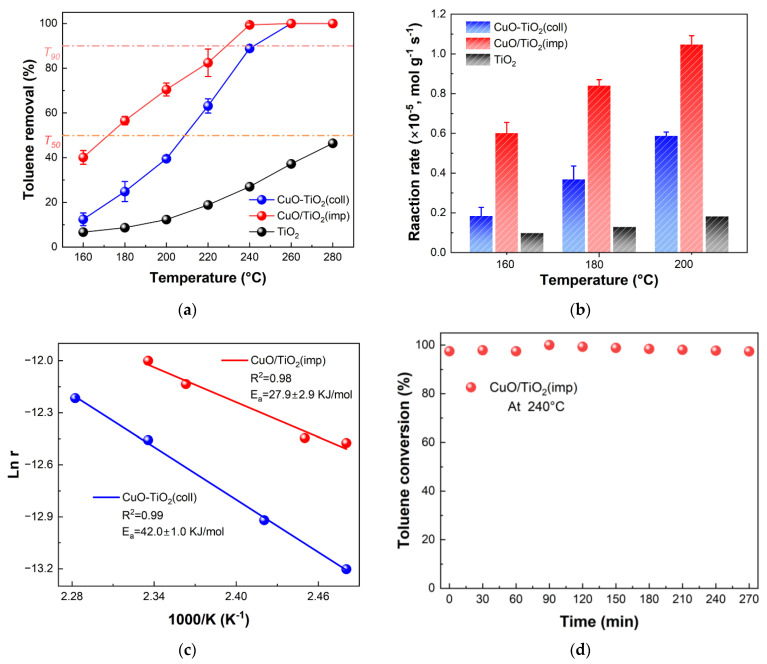
(**a**) Catalytic activity in terms of toluene conversion of catalysts; (**b**) normalized reaction rates of samples for toluene catalytic oxidation at 160 °C, 180 °C and 200 °C; (**c**) Arrhenius plot and (**d**) stability test of CuO/TiO_2_(imp) at 240 °C.

In order to study the catalytic performance, the obtained catalysts were tested in the combustion abilities of 600 ppm toluene at a space velocity of 20,000 mL·h^−1^·g^−1^. Pure TiO_2_ was tested for comparison. The temperature of 50% and the 90% removal rate of toluene (T_50_ and T_90_) are usually used to compare the low-temperature performance of the catalysts. As can be seen in Figure 1a, both CuO-TiO_2_(coll) and CuO/TiO_2_(imp) catalysts have good catalytic oxidation activity for toluene. The quantified activity data of different samples (T_50_ and T_90_) are shown in increasing order: CuO/TiO_2_(imp) (T_50_ = 170 °C, T_90_ = 225 °C) < CuO-TiO_2_(coll) (T_50_ = 210 °C, T_90_ = 240 °C) < TiO_2_. Obviously, CuO/TiO_2_ has the lowest ignition temperature and the best toluene degradation activity.

In Figure 1b, the difference in catalyst activity is further verified by calculating the normalized reaction rate. It is found that the reaction rate of toluene oxidation at 160 °C over CuO/TiO_2_(imp) is up to 6.4 × 10^−6^ mol·g^−1^·s^−1^, which are almost four and six times higher than CuO-TiO_2_(coll) (1.5 × 10^−6^ mol·g^−1^·s^−1^) and TiO_2_ (1.0 × 10^−6^ mol·g^−1^·s^−1^), respectively. A similar trend is observed at 180 °C and 200 °C. These results confirm that CuO/TiO_2_(imp) has superior catalytic activity. In addition, the performance of some copper-based catalysts for catalytic oxidation of toluene in recent years are summarized for comparison (Table 1). CuO/TiO_2_(imp) displays relatively satisfactory catalytic performance on toluene combustion with a lower T_50_ compared to the reported various transition metal oxide catalysts.

To further study the difference in catalytic activity, kinetic analysis was conducted, and an Arrhenius plot was depicted, as shown in Figure 1c. The apparent activation energy (E_a_) was calculated, and the results are listed in Table 2. The activation energy follows the following sequence: CuO/TiO_2_(imp) (27.9 ± 2.9 kJ·mol^−1^) < CuO-TiO_2_(coll) (42.0 ± 1.0 kJ·mol^−1^). It was found that the apparent activation energy (Ea) was consistent with the order of the catalytic performance. The CuO/TiO_2_ obtained lower activation energy, indicating the catalyst could activate the toluene molecules at a lower temperature [25], which was consistent with the catalytic activity. The stability of the catalysts over time is crucial for practical applications. Therefore, the stability of CuO/TiO_2_(imp) was evaluated at 240 °C (WHSV = 20,000 mL·g^−1^·h^−1^). As shown in Figure 1d, the catalyst activity remained at around 98% within 270 min.

### 3.2. Textural Properties

**Figure 2:**
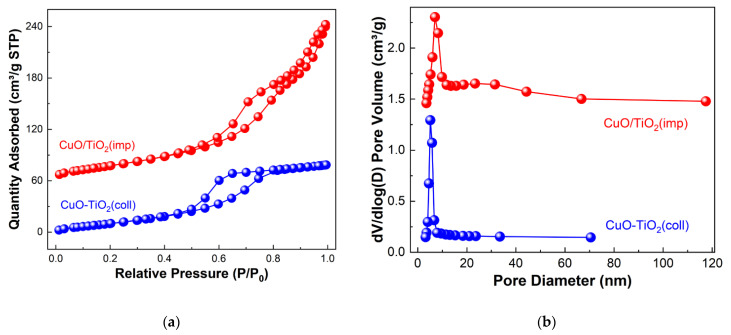
(**a**) N_2_ adsorption–desorption isotherm and (**b**) BJH pore-size distributions of samples.

N_2_ physical adsorption–desorption experiment was applied to study the textural properties of the samples. The profiles are shown in Figure 2. According to IUPAC classification, it can be seen that both CuO-TiO_2_(coll) and CuO/TiO_2_(imp) show a typical type-IV isotherm, which is suggestive of the existence of the mesoporous pore structure [12]. The difference is that a H2-model hysteresis loop appeared on CuO-TiO_2_(coll), whereas a H3-model hysteresis loop appeared on CuO/TiO_2_(imp) (Figure 2a). The pore size distribution of the samples is shown in Figure 2b. The mean pore size of CuO-TiO_2_(coll) was around 6 nm, while that of CuO/TiO_2_(imp) covered a wide range of 7–30 nm, with an average pore size of 10 nm (Table 2). Additionally, the total pore volume of CuO/TiO_2_(imp) (0.32 cm^3^·g^−1^) is twice than that of CuO-TiO_2_(coll) (0.16 cm^3^·g^−1^), which could suggest that CuO/TiO_2_(imp) has a significant toluene adsorption capacity [35]. Compare to CuO-TiO_2_(coll) (67 m^2^·g^−1^), the BET surface area of CuO/TiO_2_(imp) (97 m^2^·g^−1^) increased by about 50%. It is widely reported that the catalysts with a large specific surface area could facilitate the dispersion of the active species and transportations of reactants [36]. From this point, Cu component may be highly dispersed over TiO_2_ and, thus, contribute to its considerable catalytic performance.

### 3.3. Structural Analysis

**Figure 3:**
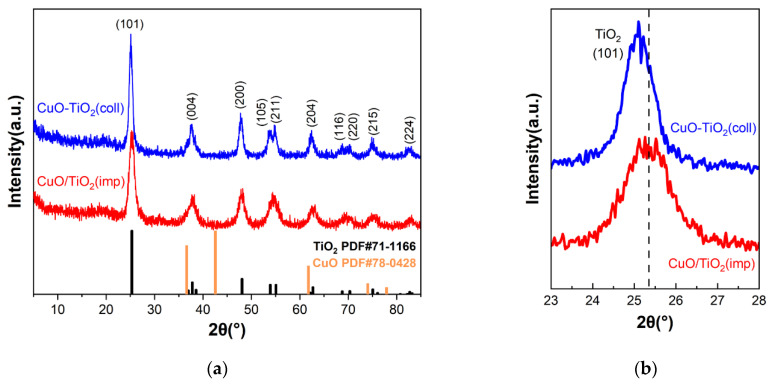
(**a**) XRD patterns of CuO-TiO_2_(coll) and CuO/TiO_2_(imp) catalysts and (**b**) partially magnified profiles.

The structure of the samples was investigated using powder XRD measurements, and the results are shown in Figure 3. For both catalysts, it can be clearly seen that the diffraction peaks are similar (Figure 3a) and can distinguish the peaks at 2θ = 25.3°, 37.8°, 48.1°, 55.1°, 62.8°, 75.2° and 82.7°, which correspond to (101), (004), (200), (211), (204), (215) and (224) crystal planes of anatase crystal TiO_2_ (PDF#71-1166), respectively. Among them, the crystal plane with the strongest diffraction peak is (101) crystal plane. Compared to CuO/TiO_2_(imp), the diffraction peak of CuO-TiO_2_(coll) shifts to a smaller diffraction angle (Figure 3b), which may be due to the different interaction between Cu and Ti in the two catalysts affecting the structure. The diffraction peak of CuO-TiO_2_(coll) is sharper than that of CuO/TiO_2_(imp), and the peak strength is stronger, indicating that CuO-TiO_2_(coll) catalyst has larger grain size and higher crystallinity. However, the diffraction peaks of Cu species are not detected, which may be due to the good dispersion of Cu or the possibility of forming amorphous or nanocrystalline metals that are too small to be detected via XRD [37].

**Figure 4:**
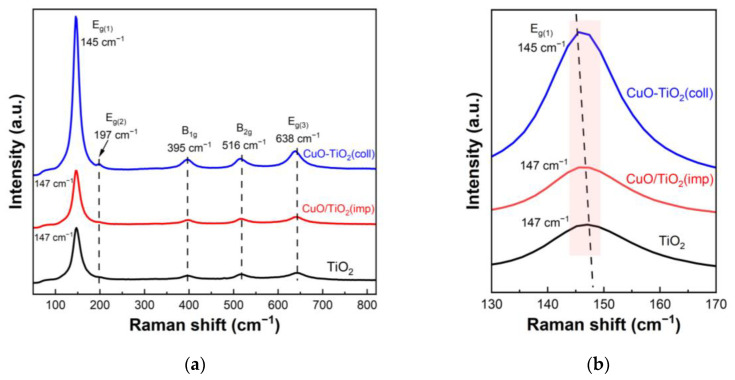
(**a**) Raman spectra of catalysts and (**b**) partially magnified profiles.

Raman spectroscopy was further used to detect the structural differences between samples. Figure 4 shows the Raman spectra of the samples. The Raman spectrum of the CuO-TiO_2_(coll) shown in Figure 4a exhibits five pronounced peaks at 145 cm^−1^ (E_g(1)_), 197 cm^−1^ (E_g(2)_), 395 cm^−1^ (B_1g_), 516 cm^−1^ (B_2g_) and 638 cm^−1^ (E_g(3)_), which are ascribed to the anatase phase of the TiO_2_, as reported in the literatures [38,39]. The Raman spectrum of CuO/TiO_2_(imp) also appears at similar peaks, indicating the typical anatase TiO_2_ phase. Additionally, no peak of Cu species is found in the pattern, which may be due to the well-dispersed CuO; this result is also consistent with XRD results. The amplified Raman spectra are shown in Figure 4b, where it can be seen that there is a slight shift in the position of Raman peaks. We note that a red shift is observed for the CuO-TiO_2_(coll) (145 cm^−1^) compared to CuO/TiO_2_(imp) (147 cm^−1^), which might be associated with the interaction between CuO and TiO_2_ [39,40,41]. Compared to Pure TiO_2_, CuO-TiO_2_(coll) has obvious migration, while CuO/TiO_2_(imp) is similar to pure TiO_2_; this outcome indicates that the interaction in CuO-TiO_2_(coll) is strong while that in CuO/TiO_2_(imp) is weak. Therefore, the structure of the sample is affected, resulting in the difference in catalytic activity.

**Figure 5:**
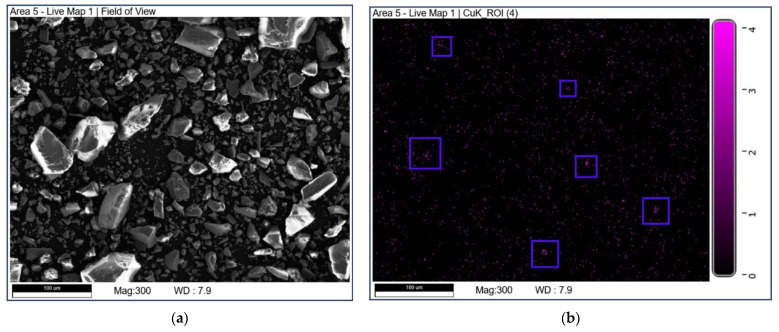

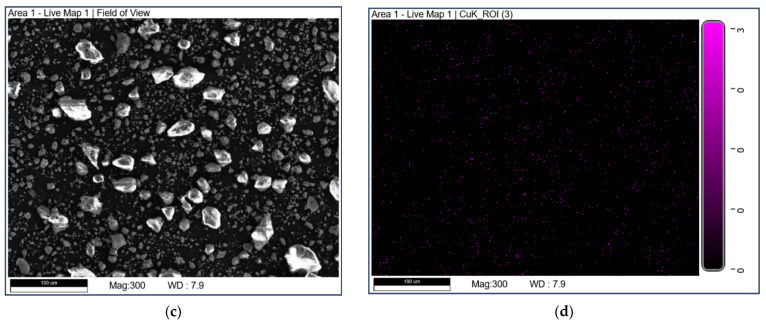
SEM-EDX mapping of Cu over (**a**,**b**) CuO-TiO_2_(coll) and (**c**,**d**) CuO/TiO_2_(imp).

SEM-EDX mapping was used to study the elemental distribution on the catalyst surface. The mapping diagrams of Cu are shown in Figure 5. It can be seen that the part marked by a blue box in Figure 5b may be due to the uneven distribution of Cu elements on the surface of CuO-TiO_2_(coll). In comparison, Copper species are well dispersed on CuO/TiO_2_(imp).

**Figure 6:**
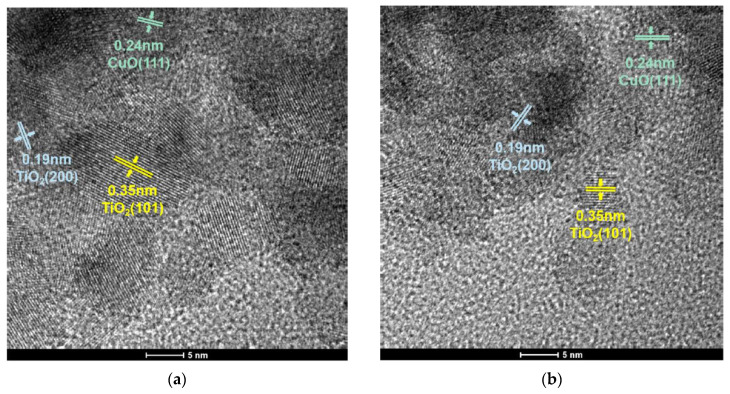

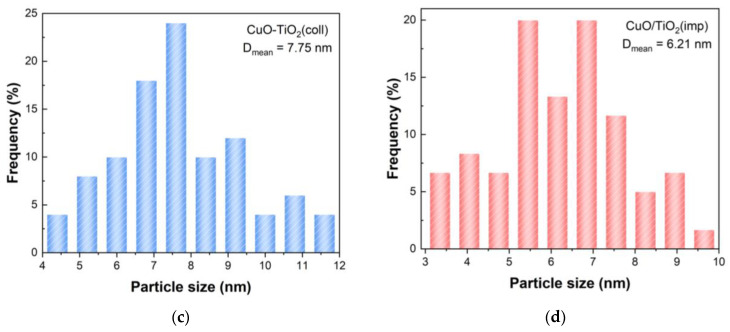
TEM images of (**a**) CuO-TiO_2_(coll) and (**b**) CuO/TiO_2_(imp) catalysts; grain particle sizes distribution of (**c**) CuO-TiO_2_(coll) and (**d**) CuO/TiO_2_(imp) catalysts.

The morphology of the catalyst was characterized via transmission electron microscopy (TEM). The TEM images are shown in Figure 6a,b. It can clearly be seen that both catalysts have clear lattice stripes, and the distances were 0.35 nm, 0.19 nm and 0.24 nm, corresponding to the TiO_2_ (101), TiO_2_ (200) and CuO (111), respectively. The grain sizes of samples were estimated using TEM images, and the average grain particle size distribution are presented in Figure 6c,d. The mean particle size of CuO-TiO_2_(coll) and CuO/TiO_2_(imp) are 7.75 nm and 6.21 nm, respectively (Table 2).

### 3.4. Characterization of Surface Element Distribution 

**Figure 7:**
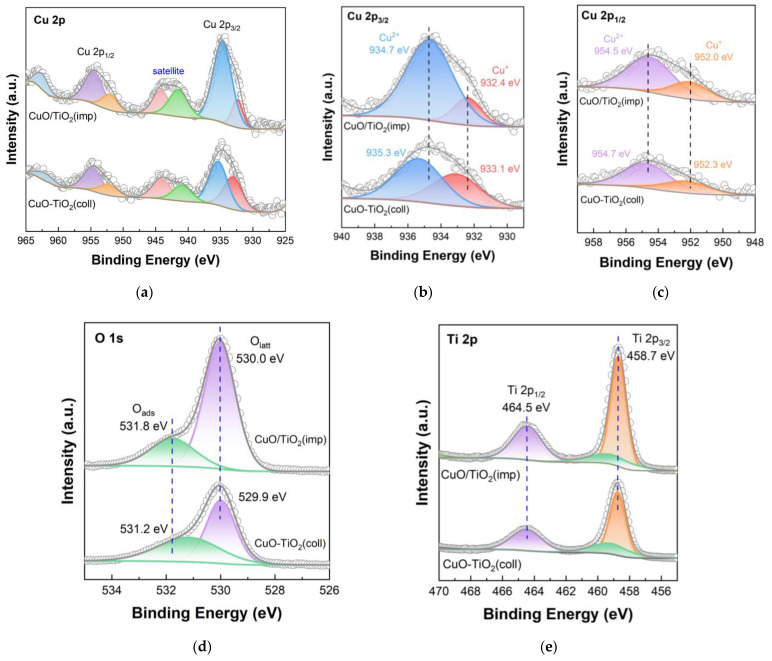
XPS spectra of catalysts: (**a**) Cu 2p; (**b**) partially magnified profile of Cu 2p_3/2_; (**c**) partially magnified profile of Cu 2p_1/2_; (**d**) O 1s; (**e**) Ti 2p.

XPS technique was used to study the element state and surface element distribution, and the results are shown in Figure 7. The spectrum of Cu is shown in Figure 7a with a range of 925 to 965 eV. The well-defined peaks of Cu 2p are determined at 938–931 eV and 958–950 eV from the XPS results, which are related to Cu 2p_3/2_ and Cu 2p_1/2_, respectively [37]. The peaks at 947–938 eV and 965–960 eV belong to satellite peaks of the Cu 2p [42,43]. According to Figure 6c and Figure 7b, it can be seen that Cu^+^ and Cu^2+^ coexist on the surface of the catalysts. Two peaks around 932 and 952 eV could be attributed to Cu^+^, while the peaks near 935 and 954 eV are assigned to Cu^2+^ [26,44]. The Cu^2+^ binding energy (935.3 eV) of the CuO-TiO_2_(coll) is slightly higher than that of CuO/TiO_2_(imp) (934.7 eV); according to previous study on copper oxide based catalysts, the higher binding energy (BE) is possibly caused by the interaction between TiO_2_ support and active component, leading to the shift of the Cu binding energy [45]. The Cu^+^ BE shows similar migration results (from 933.1 eV to 932.4 eV), which further confirms the different Cu–Ti interaction in the catalysts. 

Combined with XRD and Raman results, it could be determined that different Cu–Ti interactions exist in CuO-TiO_2_(coll) and CuO/TiO_2_(imp) samples, and the interaction between Cu and Ti for the CuO-TiO_2_(coll) sample may be stronger. Cu^2+^/(Cu^+^ + Cu^2+^) atomic ratios are also listed in Table 3. Compared to CuO-TiO_2_(coll) (0.6), the ratio of Cu^2+^/(Cu^+^ + Cu^2+^) of CuO/TiO_2_(imp) (0.8) increases up to nearly 30%. According to the above conclusions, different interactions affect the dispersion of CuO on the surface and, thus, the surface Cu content. Moreover, the reduction in the Cu^2+^/(Cu^+^ + Cu^2+^) atomic ratio of CuO-TiO_2_(coll) further confirms that the Cu–Ti interaction is stronger. As the literature previously reported, Cu^2+^ component is generally considered the active species for catalytic toluene oxidation [18] and might promote the activity of catalytic oxidation of toluene [34]. Thus, CuO/TiO_2_(imp) would show accepted catalytic activity, which is in line with the results of catalytic performance testing.

Figure 7d shows the O 1s orbital energy spectrum of the catalyst. Through peak fitting, O 1s is composed of two overlapping peaks, indicating that there are two different types of oxygen on the catalyst surface. The two peaks are located at around 530.0 eV (529.9 eV) and 531.8 eV (531.2 eV), which correspond to surface lattice oxygens (O_latt_) and surface adsorbed oxygen species (O_ads_) [46,47], respectively. The O_latt_/(O_latt_ + O_ads_) radios of both catalysts are listed in Table 3. The O_latt_/(O_latt_ + O_ads_) ratios of CuO/TiO_2_(imp) (0.81) is slightly higher than that of CuO-TiO_2_(coll) (0.77). According to previous studies [48], the toluene could be converted to CO_2_ and H_2_O by means of lattice oxygen. Therefore, the CuO/TiO_2_(imp) catalyst exhibits the higher O_latt_/(O_latt_ + O_ads_) ratio, which is beneficial for the toluene catalytic oxidation.

Figure 7e shows the XPS diagram of the Ti 2p orbital, in which the BE values of Ti 2p_1/2_ and Ti 2p_3/2_ are shown as about 464.5 eV and 458.7 eV, respectively, and there is a shoulder peak of Ti 2p_3/2_ at 459.6 eV. This finding indicates that Ti species exist in the form of Ti^4+^, rather than in the form of Ti^3+^ [49,50]. In comparison, it is found that the electron-binding energy of Ti 2p orbital of CuO-TiO_2_(coll) and CuO/TiO_2_(imp) catalysts are almost the same, while the location of the characteristic peak does not shift, which may indicate that the crystal structure of TiO_2_ was not affected.

### 3.5. Optical Properties

**Figure 8:**
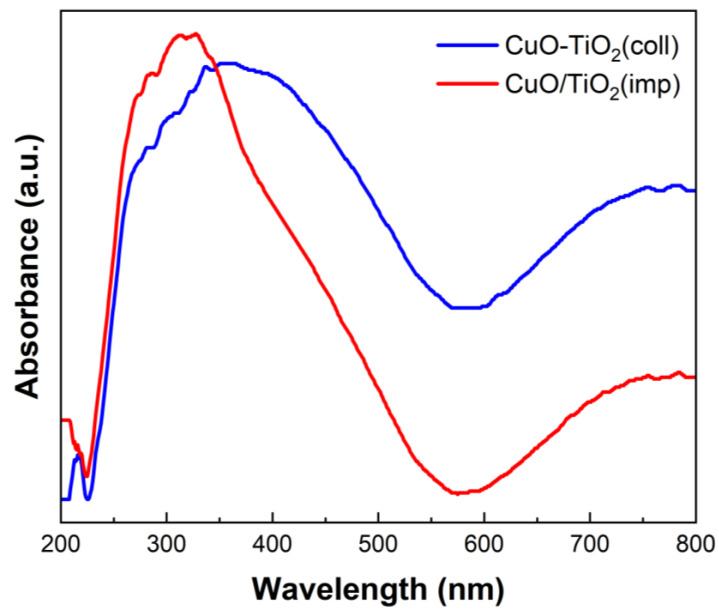
UV-vis spectroscopy of different catalysts.

UV-visible spectroscopy was used to characterize the optical properties of the materials and study the interaction between CuO and TiO_2_. The absorption spectrum is shown in Figure 8. Corresponding to the previous report [51], the absorption bands in the range of 200–230 nm are caused by the migration of lattice O^2−^ to isolated Cu^+^ or Cu^2+^ ions. Moreover, the absorption bands within 600–800 nm are assigned to the d-d transition of Cu^2+^ [42]. Additionally, the absorption band in the middle (280–570 nm) is associated with TiO_2_ [40,52]. The adsorption band intensity at 350–800 nm of CuTi samples decreased in the order CuO-TiO_2_(coll) > CuO/TiO_2_(imp). The degree of interaction between the CuO and the TiO_2_ of the catalyst is correlated with the UV-visible absorption intensity [53]. Hence, the interaction between Cu and Ti in CuO/TiO_2_(imp) is weaker than that in CuO-TiO_2_(coll), which is consistent with the results of XRD, Raman and XPS.

### 3.6. Reducibility Studies

**Figure 9:**
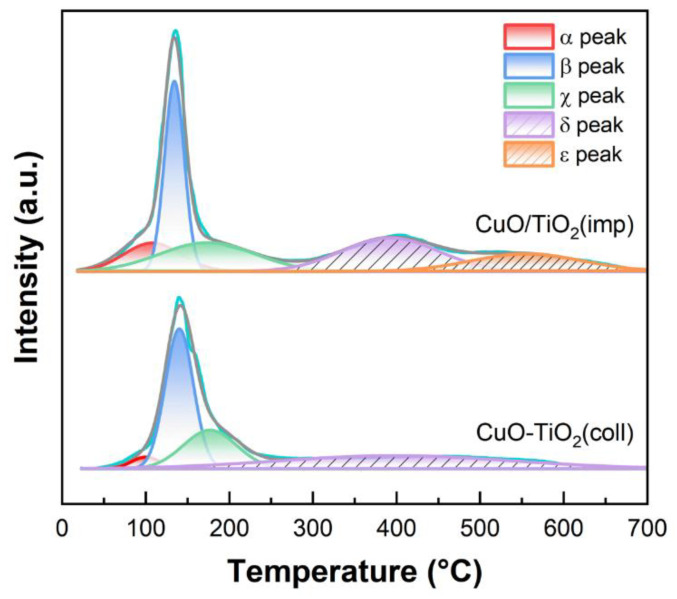
H_2_-TPR profiles of samples.

To investigate the reducibility of the samples, H_2_-TPR measurement was conducted. The profile is shown in Figure 9. In the low-temperature region (<300 °C), there are three reduction peaks for both CuO-TiO_2_(coll) and CuO/TiO_2_(imp), namely α, β and χ. α peak (20–180 °C) is attributed to the reduction in highly dispersed CuO, while β peak (100–180 °C) is ascribed to the reduction in strongly interacting CuO with TiO_2_ support [37,54]. The peak at a higher temperature (denoted as χ peak) could be assigned to the reduction in relatively larger CuO particles or bulk CuO [54]. In addition, two reduction peaks over 300 °C are correlated with TiO_2_, which are denoted by δ and ε, respectively. According to the literatures [50,55,56], they could correspond to the reduction in lattice oxygen species of TiO_2_. 

To gain insight into the redox performance, hydrogen consumption was calculated using CuO as internal standard (Table 4). The reduction temperature of α peak over the two samples is almost the same (100 vs. 107 °C). However, the H_2_ uptake toward α peak over CuO/TiO_2_(imp) (0.8 μmol·g^−1^) is remarkably higher than that over CuO-TiO_2_(coll) (0.16 μmol·g^−1^). This finding indicates plentiful reducible oxygen species [56,57] associated with abundant well-dispersed CuO species in the former sample, which contributes to its superior catalytic performance. In addition, the β peak temperature of CuO/TiO_2_(imp) is 11 °C lower than that of CuO-TiO_2_(coll), which also indicates that the interaction between CuO and TiO_2_ in the CuO/TiO_2_(imp) is weak, making oxygen species easier to reduce.

## 4. Conclusions

In summary, CuO-TiO_2_(coll) and CuO/TiO_2_(imp) with different metal–support interactions were prepared to catalyze toluene oxidation at low temperatures (600 ppm toluene, WHSV = 20,000 mL·g^−1^·h^−1^). Various characterization results show that the CuO/TiO_2_(imp) catalyst has plentiful Cu^2+^ active species and a larger specific surface area, which could promote the dispersion of active species on the catalyst surface. Additionally, the result of H_2_-TPR indicates abundant reducible oxygen species associated with well-dispersed CuO species in the CuO/TiO_2_(imp) sample. Moreover, according to the results of XRD, Raman, XPS, UV-vis and H_2_-TPR, there is strong Cu–Ti interaction in the CuO-TiO_2_(coll) sample, while the interaction in CuO/TiO_2_(imp) is weak, which is one of the main reasons for the activity difference. Therefore, CuO/TiO_2_(imp) has higher catalytic oxidation activity at lower temperatures, with a 90% toluene conversion temperature of 225 °C, as well as a higher normalized reaction rate (6.4 × 10^−6^ mol·g^−1^·s^−1^ at 160 °C) and lower apparent activation energy value (27.9 ± 2.9 kJ·mol^−1^), which have certain potential for practical application. This work will help researchers to design highly efficient low-temperature catalysts for the degradation of VOCs.

## Figures and Tables

**Table 1:** Summary of toluene catalytic oxidation using several copper-based catalysts.

Catalyst	Reaction Mixture	T_50 (°C)_	T_90 (°C)_	Ref.
Cu_1_Mn_1_O	1000 ppm toluene, 60 mL·min^−1^ WHSV: 20,000 mL·g_cat_^−1^·h^−1^	206	214	[26]
Cu_6_Ce_4_O_x_	1000 ppm toluene, 60 mL·min^−1^, WHSV: 36,000 mL·g_cat_^−1^·h^−1^	240	260	[27]
Cu-SmMn_2_O_5_	1000 ppm toluene, 200 mL·min^−1^, WHSV: 40,000 mL·g_cat_^−1^·h^−1^	210	222	[28]
Cu_1_Mn_1_	1000 ppm toluene, WHSV: 10,000 mL·g_cat_^−1^·h^−1^	226	229	[29]
20 wt.% CuO-TiO_2_	300 ppmv toluene WHSV: 36,000 mL·g_cat_^−1^·h^−1^	182	220	[30]
CuMn_2_O_4_-EG-350 (ethanol:glycol = 3:1)	1000 ppm toluene, 66.7 mL·min^−1^, WHSV: 20,000 mL·g_cat_^−1^·h^−1^	202	218	[31]
Cu_0.2_Co	1000 ppm toluene, 100 mL·min^−1^, WHSV: 40,000 mL·g_cat_^−1^·h^−1^	216	238	[32]
Cu_1_Mn_2_Ce_4_	5.0 g/m^3^ toluene 200 mL·min^−1^, WHSV: 24,000 mL·g_cat_^−1^·h^−1^	213	218	[33]
Cu_1_Ce_3_	1000 ppm toluene, 150 mL·min^−1^, WHSV: 30,000 mL·g_cat_^−1^·h^−1^	167	178	[34]
CuO/TiO_2_	600 ppm toluene, 100 mL·min^−1^, WHSV: 20,000 mL·g_cat_^−1^·h^−1^	170	225	This work

**Table 2:** BET Surface Area (m²∙g^−1^), total pore volume (cm^3^·g^−1^), average pore size (nm), average grain sizes and activation energy (E_a_) of CuO-TiO_2_(coll) and CuO/TiO_2_(imp) catalyst investigated.

Sample	BET Surface Area (m²∙g^−1^) ^a^	Total Pore Volume (cm^3^·g^−1^) ^a^	Average Pore Size (nm) ^a^	Average Grain Sizes (nm) ^b^	E_a_ (KJ∙mol^−1^) ^c^
CuO-TiO_2_(coll)	67	0.16	6.4	7.75	42.0 ± 1.0
CuO/TiO_2_(imp)	97	0.32	10.1	6.21	27.9 ± 2.9

^a^ From results of N_2_ physical adsorption–desorption experiment. ^b^ Obtained from TEM results. ^c^ From kinetic analysis results.

**Table 3:** Summary of binding energy (BE) and atomic radios of catalysts.

Samples	Cu^+^ BE/eV	Cu^2+^ BE/eV	Cu^2+^/(Cu^+^ + Cu^2+^)	O_latt_/(O_latt_ + O_ads_)	Ti^4+^ BE/eV
CuO-TiO_2_(coll)	933.1	935.3	0.61	0.77	458.7/464.5
CuO/TiO_2_(imp)	932.4	934.7	0.80	0.81	458.7/464.5

**Table 4:** Relative intensity of reduction peaks and peak temperature on H_2_-TPR curves.

Sample	α Peak	β Peak	χ Peak	δ Peak	ε Peak	Total
CuO-TiO_2_(coll)	0.16 ^a^/100 °C ^b^	1.94 ^a^/143 °C ^b^	1.01 ^a^/177 °C ^b^	1.5 ^a^/399 °C ^b^	- ^c^	4. 61 ^a^
CuO/TiO_2_(imp)	0.8 ^a^/107 °C ^b^	1.84 ^a^/132 °C ^b^	1.35 ^a^/173 °C ^b^	1.59 ^a^/392 °C ^b^	0.97 ^a^/555 °C ^b^	6. 55 ^a^

^a^ H_2_ consumption (μmol·g^−1^) calculated from H_2_-TPR results. ^b^ Peak temperature (°C). ^c^ Represented as not being detected.

## Data Availability

The authors declare that they have no known competing financial interests or personal relationships that could appear to influence the work reported in this paper.
